# Red induces hyperalgesia and white induces hypoalgesia regardless of pain modality

**DOI:** 10.1038/s41598-023-33313-w

**Published:** 2023-04-19

**Authors:** Karolina Wiercioch-Kuzianik, Justyna Brączyk, Helena Bieniek, Przemysław Bąbel

**Affiliations:** Pain Research Group, Institute of Psychology, Jagiellonian University, ul. Ingardena 6, 30-060 Kraków, Poland

**Keywords:** Psychology, Human behaviour, Colour vision, Pain

## Abstract

Colors are an important factor that influences different aspects of people's lives. However, little is known about the effects of colors on pain. This preregistered study aimed to investigate whether the type of pain affects the impact of colors on pain intensity. 74 participants were randomly divided into 2 groups according to the type of pain: electrical or thermal. In both groups, pain stimuli of the same intensity were preceded by different colors. Participants rated the pain intensity induced by each pain stimulus. Additionally, pain expectations related to each color were rated at the beginning and the end of the procedure. A significant effect of color on pain intensity ratings was found. Pain was most intense in both groups after red, whereas the lowest ratings were given after white. A similar pattern of results was observed for pain expectations. Expectations also correlated with and were found to be a predictor of experienced pain for white, blue, and green. The study shows that white can reduce, while red can alter the experienced pain. Moreover, it shows that the effect of colors is affected to a greater extent by the pain expectations rather than the pain modality. We conclude that the way colors influence pain broadens the current knowledge on effects of colors on human behavior and could help in the future both patients and practitioners.

## Introduction

Colors have been proven to influence many aspects of life. Color affects cognitive functions such as attention^1^, behavior, including consumption choices^2^ and emotional states^3^. It can convey specific meaning and the associations are not only innate but also learnt through experience and influenced by physical and psychological contexts of the situation in which the color is perceived^4^. Studies on color perception have shown that people tend to associate red with negative emotions and danger (blood, fire, anger)^5^ as in everyday life red is used to convey negative information (e.g. traffic lights, sirens, alarms). On the other hand, green is associated with nature and creates a feeling of comfort^6^. It has been proven to enhance creativity^7^ and to have a beneficial effect on mental health^8^. Also, cool colors (e.g. green or blue) have been found to elicit more pleasure and relaxation than warm colors^9^.

What is more, colors have been proven to affect pain. Red has been found to increase pain intensity more than green and blue when electrical pain stimuli are used; however, of all colors examined (red, blue, green, orange, yellow and pink), only green was found not to induce hyperalgesia when compared to no color condition^10^. When thermal stimuli are used, pain preceded by red serving as a cue for “hot” has been more intense than when preceded by blue serving as a cue for “cold”. However, the study focused on the evaluative context of the color rather than the direct effect of colors on pain^11^. Another study that examined whether the skin color would alter the heat pain threshold also found that a reddened arm significantly decreased the pain threshold compared to normal and bluish skin^12^.

The differential impact of colors on various modalities of pain could be attributed to, next to the influence of innate and learned associations and expectations, the distinct processing mechanisms involved in pain induced by electrical or thermal stimulation. Although many nociceptors respond to different stimulus modalities, some have more specialized response properties^13^. Both non-noxious and noxious heat stimuli mainly activate C-fiber (unmyelinated) nociceptors, whereas electrical stimulation signals are conducted in myelinated Aδ fibers^14^. What is more, correlations between the pain ratings induced through different modalities (heat, cold, electric and ischemic) are near zero^15^. Lötsch and colleagues^16^ also found that electrical and mechanical pain is processed differently from thermal pain. Therefore, it seems plausible that the differences between pain modalities could also be manifested in the influence of colors on pain. However, to the best of our knowledge, the influence of colors on pain intensity depending on different pain modalities has not been examined yet.

Consequently, based on the results of previous studies^10–12^ we aimed to investigate whether colors can affect pain differently depending on the pain modality. We examined whether the effect of eight color hues (blue, green, grey, orange, pink, red, white and yellow) differentiates between pain modalities (thermal, electrical). Since expectations and learning are a vital factor in pain evaluation and subjective experience of pain^17,18^, we also aimed to examine whether the effect of colors would be affected by the pain expectations regarding painful stimulation during exposure to colors. Furthermore, in a post-study questionnaire, our objective was to verify whether awareness of the aim of the study (Q1) and beliefs about the influence of colors on pain (Q2, Q3) would influence the results. The study was preregistered in the Open Science Framework [osf.io/xrznm].

## Results

**Figure 1:**
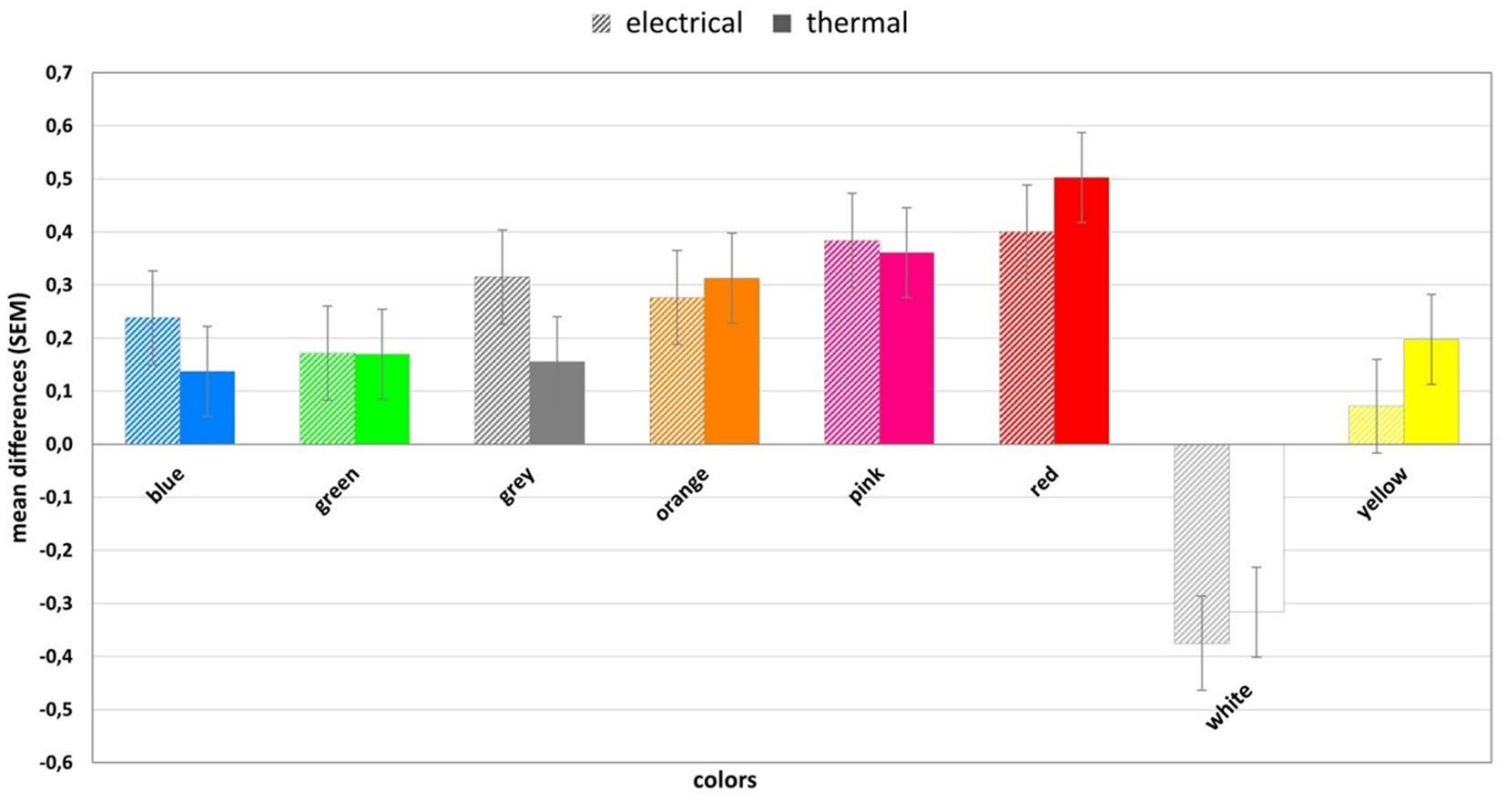
Means and standard errors of differences between pain ratings associated with black and other colors*.*

**Figure 2:**
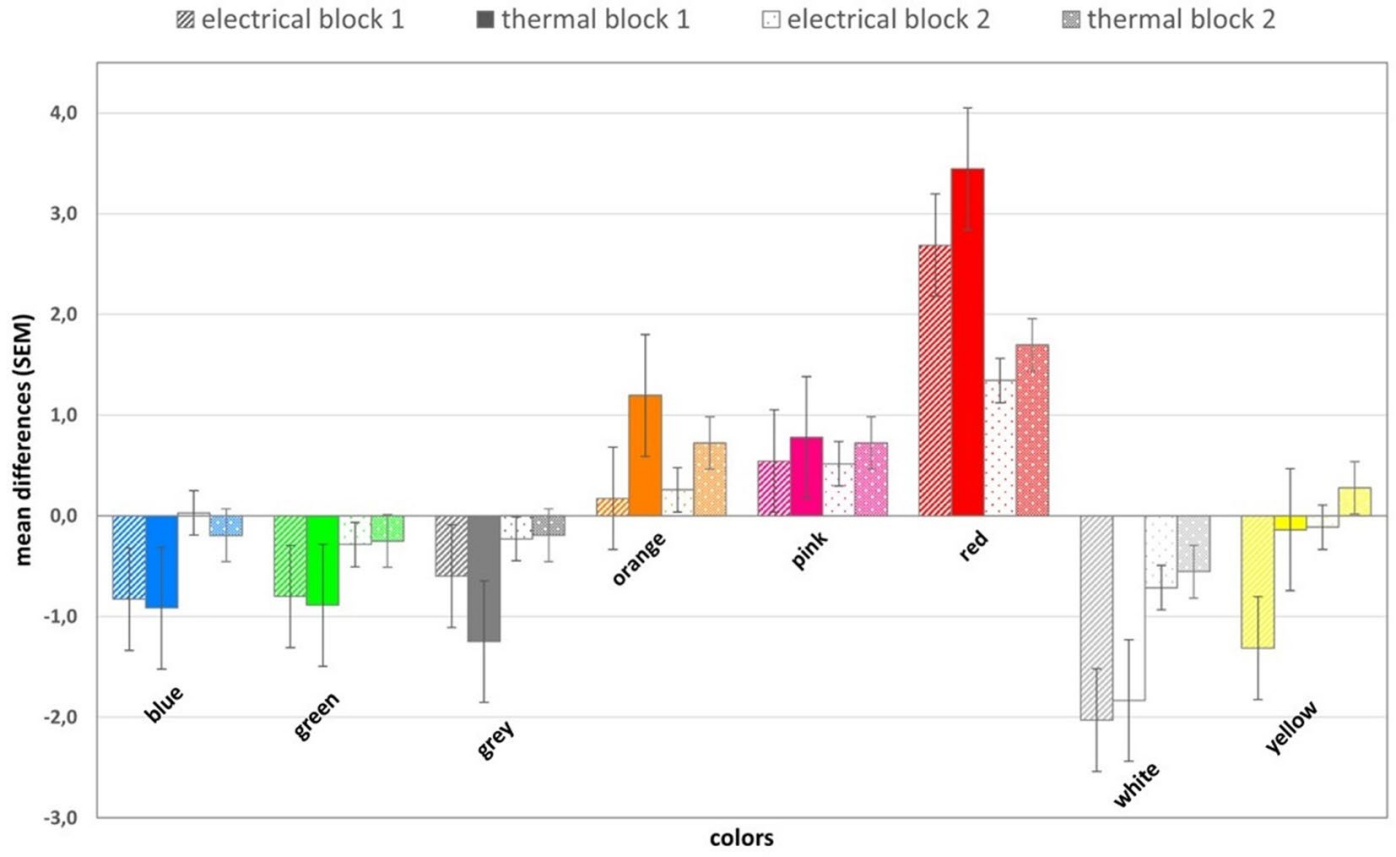
Means and standard errors of differences between expectation ratings between black and other colors.

There were no differences between groups (electrical and thermal) in terms of participants' age (*F*(1, 69) = 0.006, *p* = 0.940), BMI (*F*(1, 69) = 1.23, *p* = 0.272), sex (*X*^*2*^(2, *N* = 71) = 1.34, *p* = 0.511), education level (*X*^2^(2, *N* = 71) = 0.26, *p* = 0.880), job situation (*X*^2^(3, *N* = 71) = 0.93, *p* = 0.819), and handedness (*X*^2^(1, *N* = 71) = 0.63, *p* = 0.428). The descriptive statistics and distributions are presented in Table 1. Means and standard errors of differences between ratings associated with black and other colors are presented in Fig. 1 (pain ratings) and in Fig. 2 (expectation ratings).

**Table 1:** Basic descriptive statistics of the study participants, expressed as the mean (± standard deviation) or percentage (number) and p-values of between group analyses.

Study variables	Total sample (*N* = 71)	Thermal group (*N* = 36)	Electrical group (*N* = 35)	p-value
Numerical variables				
Age (years)	22.25 ± 3.50	22.23 ± 3.44	22.29 ± 3.61	0.940
BMI (kg/m^2^)	22.42 ± 3.35	22.86 ± 2.97	21.98 ± 3.69	0.272
Categorical variables				
Sex				
Male	38.03% (27)	41.67% (15)	34.29% (12)	0.511
Female	60.56% (43)	58.33% (21)	62.86% (22)	
Other	< 1.00% (1)	0% (0)	2.86% (1)	
Handedness				
Right	88.73% (63)	91,67% (33)	85,71% (30)	0.428
Left	11.27% (8)	8,33% (3)	14,29% (5)	
Education				
Primary school	5.63% (4)	5.56% (2)	5.71% (2)	0.880
Secondary school	71.83% (51)	69.44% (25)	74.29% (26)	
Faculty	22.54% (16)	25.00% (9)	20.00% (7)	
Job situation				
Student	76.05% (54)	80.56% (29)	71.43% (25)	0.819
Employed	12.68% (9)	11.11% (4)	14.29% (5)	
Unemployed	7.04% (5)	5.56% (2)	8.57% (3)	
Student and employed	4.23% (4)	2.77% (1)	5.71% (2)

### Pain analyses

The two-way mixed-design ANOVA performed on the pain ratings revealed a statistically significant main effect for ‘color’ (*F*(5.05, 348.54) = 22.70, *p* < 0.001, *η*^*2*^_*p*_ = 0.25), while neither the main effect for ‘modality’ (*F*(1.00, 69.00) = 0.00, *p* = 0.983, *η*^*2*^_*p*_ < 0.01) nor the ‘color’ x ‘modality’ interaction (*F*(5.05, 348.54) = 0.94, *p* = 0.455, *η*^*2*^_*p*_ = 0.01) were significant. As the sphericity assumption was violated (*p* < 0.001), the results are reported with the Greenhouse–Geisser correction.

The post-hoc tests were carried out without the modality division as there was a significant main effect for ‘color’ but not for the ‘color’ x ‘modality’ interaction. The results showed that all color comparisons which revealed significant differences consisted of either white or red (Table 2).

**Table 2:** Post-hoc differences in the pain ratings for all colors (both modalities merged).

	Blue	Green	Grey	Orange	Pink	Red	White	Yellow
Green	p = 1.000							
Grey	p = 1.000	p = 1.000						
Orange	p = 1.000	p = 1.000	p = 1.000					
Pink	p = 0.556	p = 0.199	p = 1.000	p = 1.000				
Red	p = 0.041*	p = 0.066	p = 0.181	p = 0.660	p = 1.000			
White	p < 0.001*	p < 0.001*	p < 0.001*	p < 0.001*	p < 0.001*	p < 0.001*		
Yellow	p = 1.000	p = 1.000	p = 1.000	p = 0.363	p = 0.037*	p = 0.015*	p < 0.001*	

The Bonferroni correction was used for all post-hoc tests. Significant comparisons are marked with *.

### Expectation analyses

The three-way mixed-design ANOVA that was performed on the expectation ratings showed a significant main effect for ‘color’ (*F*(4.76, 328.36) = 54.83, *p* < 0.001, *η*^*2*^_*p*_ = 0.44) and the ‘color’ x ‘block’ interaction (*F*(4.92, 339.64) = 14.62, *p* < 0.001, *η*^*2*^_*p*_ = 0.17). The main effects for ‘block’ (*F*(1.00, 69.00) = 0.51, *p* = 0.479, *η*^*2*^_*p*_ < 0.01) and ‘modality’ (*F*(1.00, 69.00) = 0.26, *p* = 0.613, *η*^*2*^_*p*_ < 0.01), as well as the ‘color’ x ‘modality’ (*F*(4.76, 328.36) = 1.88, *p* = 0.101, *η*^*2*^_*p*_ = 0.03), ‘block’ x ‘modality’ (*F*(1.00, 69.00) = 0.03, *p* = 0.867, *η*^*2*^_*p*_ < 0.01) and ‘color’ x ‘block’ x ‘modality’ (*F*(4.92, 339.64) = 0.95, *p* = 0.448, *η*^*2*^_*p*_ = 0.01) interactions were not significant. As the sphericity assumption was violated (*p* < 0.001), the results are reported with the Greenhouse–Geisser correction.

As only the main effect of ‘color’ and the ‘color’ x ‘block’ interaction effect were significant, the post-hoc tests were carried out without the modality division. The results for the first expectation block showed a lot of significant differences, mostly related to red, white, orange and pink. In the second expectation block, the list of statistically significant differences was shorter and mostly related to red, but also to some other colors (see Tables 3 and 4).

**Table 3:** Post-hoc differences in the first expectation block for all colors (both modalities merged).

	Blue	Green	Grey	Orange	Pink	Red	White	Yellow
Green	p = 1.000							
Grey	p = 1.000	p = 1.000						
Orange	p < 0.001*	p < 0.001*	p < 0.001*					
Pink	p < 0.001*	p < 0.001*	p = 0.001*	p = 1.000				
Red	p < 0.001*	p < 0.001*	p < 0.001*	p < 0.001*	p < 0.001*			
White	p = 0.003*	p = 0.008*	p = 0.230	p < 0.001*	p < 0.001*	p < 0.001*		
Yellow	p = 1.000	p = 1.000	p = 1.000	p < 0.001*	p < 0.001*	p < 0.001*	p = 0.001*	

The Bonferroni correction was used for all post-hoc tests. Significant comparisons are marked with *.

**Table 4:** Post-hoc differences in the second expectation block for all colors (both modalities merged).

	Blue	Green	Grey	Orange	Pink	Red	White	Yellow
Green	p = 1.000							
Grey	p = 1.000	p = 1.000						
Orange	p = 0.298	p = 0.024*	p = 0.098					
Pink	p = 0.069	p = 0.002*	p = 0.024*	p = 1.000				
Red	p < 0.001*	p < 0.001*	p < 0.001*	p < 0.001*	p < 0.001*			
White	p = 0.534	p = 1.000	p = 1.000	p = 0.001*	p < 0.001*	p < 0.001*		
Yellow	p = 1.000	p = 1.000	p = 1.000	p = 0.323	p = 0.030*	p < 0.001*	p = 0.038*	

The Bonferroni correction was used for all post-hoc tests. Significant comparisons are marked with *.

### Secondary analyses

Due to the non-significant ‘color’ x ‘modality’ interaction, the correlation and regression analyses were carried out without the division into modalities. Correlation analyses revealed that expectation ratings from the first expectation block were correlated with pain ratings for the following colors: blue (*r* = 0.36, *p* = 0.002), green (*r* = 0.35, *p* = 0.003), and white (*r* = 0.40, *p* = 0.001) (see Supplementary Fig. S1). Further regression analyses indicated that the expectations were also predictors of pain for blue (*adj. R*^*2*^ = 0.12, *β* = 0.382, *p* = 0.002), green (*adj. R*^*2*^ = 0.11, *β* = 0.353, *p* = 0.003) and white *(adj. R*^*2*^ = 0.40, *β* = 0.396, *p* = 0.001).

The post-study questionnaire revealed that 62% of participants figured out the real aim of the study (Q1). Moreover, 87.3% of participants believed that colors could influence pain perception (Q2), and 57.7% declared that colors had affected their pain sensation in this study (Q3). The percentage of participants who thought that colors could influence pain perception in general as well as in this study was 56.3%. In contrast, those who thought that colors could influence pain perception in general but had not affected it in this study was 31%. The additional analyses showed that participants’ awareness of the study aim (‘color’ x ‘Q1’ interaction (*F*(7, 67) = 0.85, *p* = 0.513, *η*^*2*^_*p*_ = 0.13)), their beliefs concerning the influence of colors on pain (‘color’ x ‘Q2’ interaction (*F*(7, 67) = 0.62, *p* = 0.692, *η*^*2*^_*p*_ = 0.01)), and belief that colors influenced pain in this study (‘color’ x ‘Q3’ interaction *F*(7, 67) = 0.73, *p* = 0.608, *η*^*2*^_*p*_ = 0.01)) did not confound the results.

## Discussion

This study aimed to examine the influence of pain modality, either thermal or electrical, on the effect of colors on pain perception. Consistent with previous research^10,11^, the data showed the greatest increase in pain intensity for red. Also, red intensified pain significantly compared to blue, white and yellow. The study also showed a similar pattern for expectations. Participants expected more pain related to red than any other color, regardless of when the expectation measurement took place (at the beginning or the end of the task). Surprisingly, we also found that white was the most pain-reducing color, and participants expected it to reduce their pain perception. Although we did not observe an interaction between pain modality and the effect of colors on pain perception, it is an important finding that colors influence people’s subjective perception of pain despite the type of this pain. Heat stimulation activates predominantly unmyelinated C-fibers in contrast to electrical stimulation, which is mainly transferred via myelinated Aδ fibers^14^. However, in our study it seems that participants’ expectations were more critical for pain evaluation than the difference in the biological mechanism (activation of different types of fibers). The lack of differences between the electrical and thermal pain groups might be the result of the nature of the pain experience and the strong impact of the psychological component in the pain-evaluation process. It is already known that factors such as emotions, attention or context can alter pain perception^19–21^. Moreover, colors also convey an emotional component and have been proven to elicit both positive and negative emotions^5,9^. Thus, it is possible that the effect of colors on pain perception prevails over the type of pain.

Pain intensity and pain expectations were highest for red, despite the pain modality. Furthermore, our data revealed that white reduced pain the most compared to the other colors, and this was the only color that reduced pain against the baseline. To the best of our knowledge, this is the first time that a hypoalgesic effect of white has been found. White is associated with morality, honesty, purity, and cleanliness^22–24^. In studies on drug effectiveness^25^, white is often coupled with sedative properties, and there is also evidence that white drugs are perceived as analgesics^26^. Likewise, white is a commonly used color for painkillers and many OTC medications, which might explain the obtained results. On the other hand, drugs that are colored red are usually associated with a stimulant effect^25^, but this color is also associated with failure and general negative words^5^. In everyday life, red frequently serves as a negative information medium (e.g. traffic lights, warning signals, alarms, decrease in value) and often warns of danger, such as blood, an angry face, or fire. The hyperalgesic effect of red is also in line with previous research on the effects of colors on pain perception^10^. Moreover, the red-white spectrum is used in the Color Analog Scale (CAS)^27^, which is commonly used as a self-report measure of pain in children^28–30^ and is an alternative to the Visual Analog Scale (VAS) or the Numerical Rating Scale (NRS), both of which are commonly used with adults. CAS is a tool in which pain intensity is related to the color transition from white to red, where white represents no pain, and dark red corresponds to the highest pain. The design of the scale seems to reflect common notions and associations between different color hues (red and white) and pain. All of the mentioned aspects may indicate that colors affect pain through prior experience and the fact that people learn over the course of their lives the associations between particular colors and pain.

It is suggested that colors convey specific meaning and information and influence behavior through entrenched associations^31^. The observed hyperalgesic effect of red and the hypoalgesic effect of white might thus be linked to giving importance to these colors and one’s previous experience. An equally important factor affecting pain intensity ratings was probably the influence of the pain intensity expectations for each color. We found that blue, green, and white pain expectation ratings were predictors of pain intensity ratings. Additionally, prior to pain induction, people expected the most intense pain after exposure to red, followed by pink and orange. On the other hand, the colors perceived as reducing pain the most were white, followed by blue, green, grey, and yellow. This pattern of results was preserved over time, but the magnitude of differences between each color and the baseline decreased. At the end of the experiment, red was still perceived as the most pain increasing color. Significant differences remained between green, white, and red, orange, or pink, but for blue only the difference with red remained significant. A more thorough examination of the mean values shows that direct experience of color effects has flattened the expectations. This means that the expectation of high pain has decreased, but the expectation of low pain has increased. This is in line with the probable direction, as participants received stimuli of one, individually calibrated intensity. In the literature, expectations have been associated with modulation of the pain experience^32,33^. On the other hand, placebo research indicates that experience and conditioning can alter pain perception despite the evoked expectations^34,35^. Overall, it may be possible that participants’ initial expectations influenced their pain experience associated with different colors, but the experience itself might have further affected the subsequent expectations. It is also worth noting that the majority of participants believed that colors generally influence pain perception and declared that colors had affected their pain perception in our study. However, these beliefs did not affect the overall outcomes. Further investigation focused specifically on the role of expectations in the relationship between colors and pain perception is still needed.

The strengths of this study include the use of a mixed-method design, which enabled us to explore in depth the effect of colors on pain perception and test it against the two types of pain. As a result, we not only found that colors influence pain regardless of the modality of pain, but we were also able to replicate previous findings which showed that red increases pain more than all other colors. This study’s results clearly demonstrate that colors affect pain in humans, and the most novel finding is that white is the most hypoalgesic color. However, the findings of this study have to be seen in the light of some limitations. First, although we calibrated pain stimuli intensity to 5/10 on NRS, the average pain intensity ratings for the first baseline were 4.1 and 3.5 in the electrical and thermal groups, respectively. We observed a non-significant sensitization effect between the first and the second baselines in both groups, but the average pain intensity ratings were still lower than 5/10 (4.2 in the electrical group and 3.8 in the thermal group). Research on classical fear conditioning shows that the more painful an unconditioned stimulus, the faster an aversive emotional association is established^36,37^. Thus, the intensity of painful stimulation might be an important factor in other pain settings, such as the one presented in the current study. Second, only a healthy, pain-free population, aged between 18 and 35 years old was included in the pooled sample, thus resulting in low ecological validity. Third, the study was conducted among people from Western culture. It is known that color meaning may be culturally dependent and differences in color preferences occur^38–40^, which is why our results might not be directly translated into other cultures. Notwithstanding these limitations, it is at the same time an essential perspective for further studies.

Further investigation and broadening of the participant pool to a patient or pain-susceptible population would enable a generalization or review of the current findings. However, our results already show that there is a need for careful design of experimental and clinical protocols, as well as the data interpretation. Our findings could facilitate the methodology of other pain studies which use colors as cues or as part of a procedure (e.g. investigating chronic pain interventions or placebo effects). Additional research is needed to understand how the effect of colors manifests in other conditions (e.g. general practice care, hospital care) and how it can be implemented in clinical practice, such as pain management therapies for either chronic or acute pain. There is also a need for identification and research on mechanisms behind the effect of colors on pain and whether they are similar to some extent to mechanisms related, e.g., to open-label placebo.

## Material and methods

The study protocol was approved by the ethics committee at the Institute of Psychology, Jagiellonian University, Cracow, Poland (KE/25_2021) and was preregistered in the Open Science Framework [osf.io/xrznm]. All methods were carried out in accordance with the Declaration of Helsinki.

### Participants

A total of 124 healthy participants aged 18 to 35 years were initially recruited through advertisements on social media and job portals, and by word of mouth. The exclusion criteria were previous participation in pain experiments; being a student (3 years or more) or graduate of psychology, cognitive science, or a medical major; the presence of chronic or acute pain; alcohol abuse; having unremovable metal objects in the forearms; diagnosed neurological, cardiovascular, metabolic, musculoskeletal, or psychological disorders in the preceding six months; current drug consumption; abnormal color vision. The exclusion criteria were evaluated with an online questionnaire, and only participants eligible for the study were invited for an experimental session.

Participants were informed that the purpose of the study was to assess their pain sensitivity and how they respond to painful stimulation. They were required to provide informed consent and were financially compensated with 30PLN (~ 7USD) for completing the study. Fifty participants did not show up for the experimental session. They were compared with the 74 participants who did take part in the study in terms of BMI, education level, job situation, and sex, but no differences were found. Data from three participants were not assessed due to technical malfunctions (n = 2) and data file corruption (n = 1). Thus, the statistical analysis was performed on data set of 71 cases. The basic descriptive statistics of the tested participants are presented in Table 1.

### Sample size

The sample size calculation was conducted using G*Power 3.1 software^41^ and was based on the F-test for repeated measures ANOVA with within-between factor interaction. A minimum of 64 participants was required in order to enable detection of a small interaction effect (effect size of f = 0.14) with error probability (α) of 0.05 and power set at 95%. To ensure a sufficient power in case of any exclusions, the sample size was increased to 74 participants. The data collection stopped when the required sample size was reached.

### Stimuli and measures

When carrying out an experiment focused on color, significant elements are the participants’ color perception and the screen which displays the colors. The former was assessed via the Ishihara test during the screening phase^42^. An Eizo ColorEdge (model CG2730) screen with a built-in self-calibration sensor was used to ensure the proper color reproduction during the experimental session. A neutral greyish color on the walls combined with a screen-shielding hood prevented glare caused by ambient lighting.

To measure the effect of colors on pain perception, we used 6 different color hues: red (RGB (255, 0, 0)), green (RGB (0, 255, 0)), orange (RGB (255, 128, 0)), blue (RGB (0, 128, 255)), yellow (RGB (255, 255, 0)), and pink (RGB (255, 0, 128)). The colors were selected in such a way to examine primary and complementary colors. The saturation level was set at 100%, because the hues are most distinct at this level^43^. The brightness level was set at 50%. Besides the aforementioned hues, we also used 3 grey-scale colors: grey (RGB (128, 128, 128)), white (RGB (255, 255, 255)) and black (RGB (0, 0, 0)). Black served as the baseline color in the experiment due to the color display specification of computer monitors (to produce black, pixels are turned off), so it is the closest condition to no color. All colors were presented in full-screen mode (screen resolution 2560 × 1440, distance from participant approximately 60 cm).

Each group received pain stimuli of one modality: either electrical or thermal. Pain stimuli were delivered to the volar side of the non-dominant forearm within C5 dermatome (participants self-reported handedness). Electrical stimuli (Digitimer DS8R; Digitimer; Welwyn, Garden City, England) were square pulses applied in a sequence of three pulses (200 μs) with an ISI of 100 μs. Thermal stimuli were applied using a contact thermode, sized 30 × 30 mm (Model ATS and TSA-II; Medoc Ltd Advanced Medical System; Israel), starting from a 32 °C baseline temperature with a 10 °C/s ramp-up/ramp-down ratio and 1 s plateau. To achieve the best possible similarity in pain sensation evoked with thermal and electrical stimulation, the physical characteristics of the stimuli (i.e. duration) were adjusted and calibration procedure was applied.

Participants rated pain intensity (“How painful was the stimulation?”) and pain expectation (“How painful do you expect the stimulation to be after seeing this color?”) separately on an 11-point Numeric Rating Scale (NRS), from 0 = ‘no pain’ to 10 = ‘the most intense tolerable pain’. The NRSs were always presented on a color slide corresponding to the color used in the current trial.

The procedure was programmed using Python 3 language and PsychoPy 2021 software^44^.

### Experimental design

The experiment was based on a between-subject, repeated-measures design with between-factor modality (electrical, thermal) and within-factor color (black, blue, green, grey, orange, pink, red, white, yellow). Eligible participants were invited to an experimental session and randomly assigned to either the electrical or the thermal group. The onsite session comprised two parts: calibration and the main task.

#### Calibration

Tactile threshold, pain threshold, and pain intensity were assessed during calibration. Participants in the electrical group received stimuli in steps of 1 mA per 5 s, starting from 0 mA. In the thermal group, stimuli were delivered in steps of 0.5 °C per 5 s, the baseline temperature was set at 32 °C and the first applied stimulus was 38 °C. The 5 s interval reduces the risk of occurrence of temporal summation, since stimuli are delivered at frequency of 0.2Hz^45^. Participants reported a tactile threshold with keypress as the first non-painful tactile sensation. After detecting the tactile threshold, participants in both groups assessed the pain intensity of each stimulus on the NRS until they reached a rating of 7 or more. This sequence was then repeated. Obtained pain intensity ratings with corresponding intensities (mA or °C) were then fitted to an exponential curve. Based on the curve, the individual intensity of a pain stimulus corresponding to a rating of 5 on the NRS was calculated for each participant. In the electrical group, individual intensities ranged 3.4–92.4 mA (*M* = 24.15 mA, *SD* = 19.70), while in the thermal group, intensities ranged 41.0–51.0 mA (*M* = 47.39 °C, *SD* = 2.48).

#### Main task

**Figure 3:**
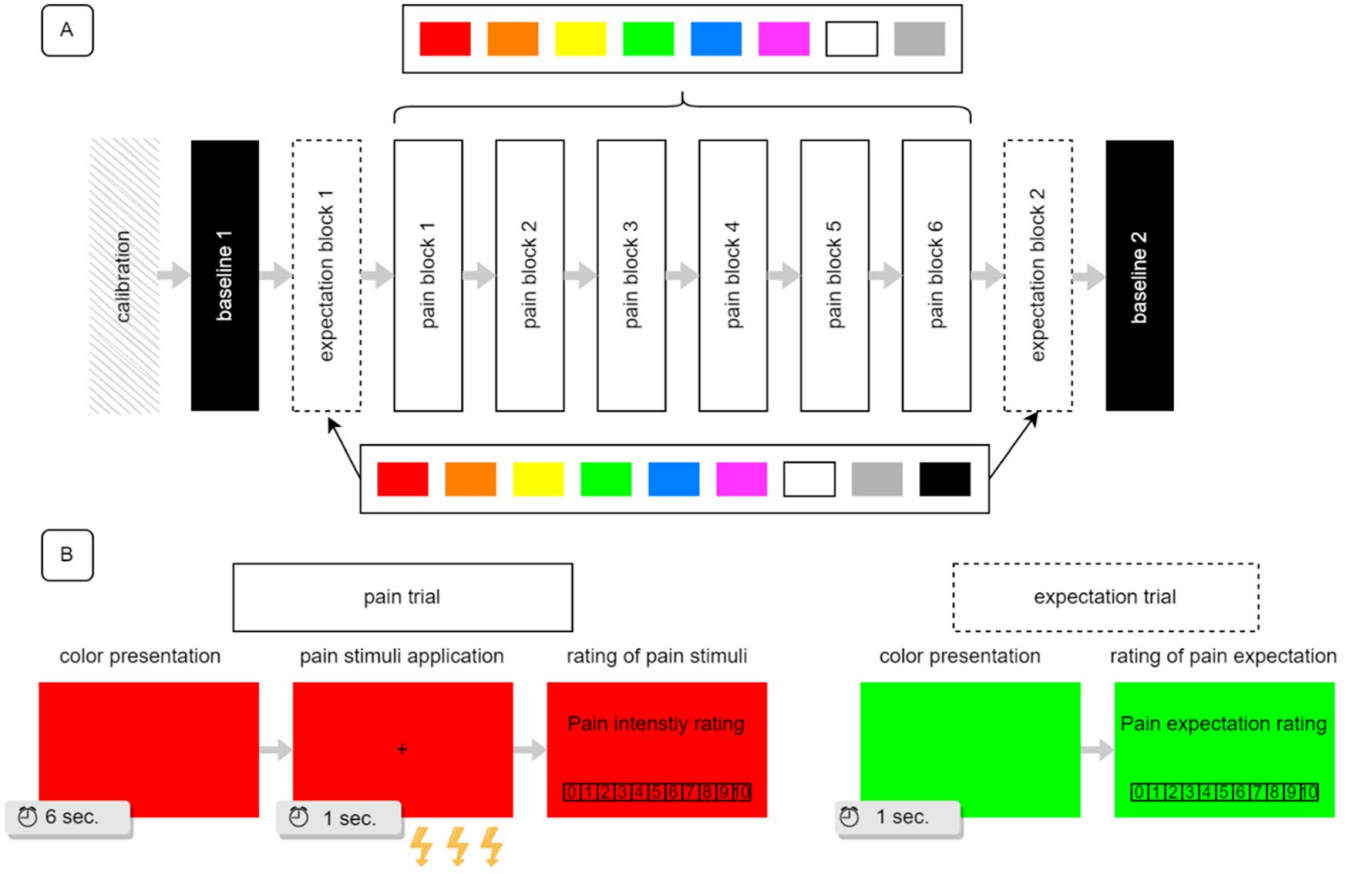
Study design. The study involved two groups that differed in the modality of pain applied: thermal or electrical. Part (**A**) of the figure shows the study flow. Part (**B**) shows a sample pain and expectation trial design.

The main task consisted of ten blocks: two pain baseline blocks, two expectation color blocks, and six pain color blocks (see the flow of the task in Fig. 3A.). In the first baseline block, only pain was measured. There were eight black trials: the first two trials served as familiarization and were not used in the analysis. In the second baseline block, there were six black trials and only pain was measured. The expectation color blocks consisted of nine trials, i.e., one trial for each color used: blue, green, grey, orange, pink, red, white, yellow, and one trial for baseline (black). In the first expectation block, black trial was always first, whereas in the second expectation block, black trial was set always last. Each pain color block consisted of eight trials (one for each color). Pain stimuli were applied only in the baseline and pain blocks (pain trials). During the pain trials, color was presented for 6 s, followed by the presentation of a fixation cross during the application of the pain stimulus (~ 1 s). After the pain stimulus was applied, the NRS for pain intensity appeared until the participant rated the sensation. Both the fixation cross and the NRS were presented on the color used in the trial. In the expectation trials, the color was presented for 1 s, after which the NRS for pain expectation appeared until the participant gave a rating (see Fig. 3B). The colors in the pain color blocks and the expectation color blocks were presented in random order.

After finishing the main task, participants were asked to fill out a questionnaire to assess whether they had figured out the aim of the study and to collect their beliefs about the influence of colors on pain perception (questions in Table 5).

**Table 5:** End of study questionnaire.

Question	Notes
Q1. In your opinion, what was the aim of the study?	Open-ended question; responses evaluated and converted to dichotomous scale (yes/no answer indicating whether participant figured out the aim of the study)
Q2. Do you think colors can influence pain perception?	Yes/no answer
Q3. In this study, were the colors related to the pain you felt?	Yes/no answer
Q4. How did the colors affect the pain you felt?	Asked only if the answer to Q3 was “yes”; participants were asked to assign each color to one of three categories: “increased the pain”, “decreased the pain”, “did not affect the pain”

### Statistical analysis

First, descriptive statistics were calculated for age and body mass index (BMI), as well as the distribution of sex, education, job situation, and handedness. This was followed by analyses of the differences between the groups (electrical and thermal), calculated using the Student’s *t* test for age and BMI, and the Chi-squared test for education level, job situation, handedness, and sex.

Then, the variables of interest were checked for outliers and prepared before the conduct of further statistical analyses: the pain ratings for each color and the baseline were aggregated into means. After, the first and the second pain baseline blocks were compared through the repeated-measures ANOVA, with ‘modality’ (electrical, thermal) as the between-subjects factor and ‘baseline’ (first, second) as the within-subjects factor. The baselines did not differ significantly, therefore they were combined into one. The baseline pain ratings were subtracted from the pain color ratings. Similarly, within both pain expectation blocks, the expectation baseline rating was subtracted from the expectation rating of every other color. Therefore, data analyses for pain and expectation were performed on the differences between the baseline (black) and the other colors.

In the primary analyses on pain intensity, a two-way, mixed-design ANOVA was used with ‘modality’ (electrical, thermal) as the between-subjects factor and ‘color’ (differences between the baseline and each of the following: blue, green, grey, orange, pink, red, white, and yellow) as the within-subjects factor. Three additional ANOVAs of the same structure were conducted with respective additional factors: ‘Q1’ (yes, no), ‘Q2’ (yes, no), or ‘Q3’ (yes, no), in order to verify whether awareness of the study aim (Q1) and beliefs about the influence of colors on pain (Q2 and Q3) influenced the results.

Also, a three-way, mixed-design ANOVA was performed on participants’ NRS expectation ratings. The ‘modality’ (electrical, thermal) was the between-subjects factor, while ‘color’ (differences between the black and each of the following: blue, green, grey, orange, pink, red, white and yellow) and ‘block ‘(first expectation block, second expectation block) served as the within-subject factors.

The post-hoc tests were performed for the pain and expectation ratings to explore between-colors differences. Correlation and regression analyses were performed to explore the relationship between pain expectation related to colors at the beginning of the experiment and further pain intensity ratings.

The alpha level was set at 0.05 for rejection of the null hypothesis. The Bonferroni correction was implemented for all analyses. The analyses were conducted using the IBM SPSS Statistics environment, version 26.0 (IBM Corp. 2019).

## Supplementary Information

Supplementary Figure S1.

## Data Availability

The data set used in analyses is available at OSF at https://osf.io/x64jr/ (https://doi.org/10.17605/OSF.IO/X64JR). The conducted research was preregistered with a brief analysis plan and is available at https://osf.io/xrznm (https://doi.org/10.17605/OSF.IO/XRZNM). The data collection started by the time of preregistration; however, none of the data has been analyzed or looked at yet until the study ended and whole sample was collected. Moreover, the study protocol was described in the ethics committee proposal before starting the experiment.
