# Supplementary material for: Red induces hyperalgesia and white induces hypoalgesia regardless of pain modality
Source: Sci Rep. 2023 Apr 19;13:6360. doi: 10.1038/s41598-023-33313-w (PMC10115883; doi:10.1038/s41598-023-33313-w)
Supplement: Supplementary file 1 — Supplementary Figure S1. [file 41598_2023_33313_MOESM1_ESM.docx]

**Red induces hyperalgesia and white induces hypoalgesia regardless of pain modality**

Karolina Wiercioch-Kuzianik^1^*, Justyna Brączyk^1^, Helena Bieniek^1^, Przemysław Bąbel^1^

^1^Pain Research Group, Institute of Psychology, Jagiellonian University, Kraków, Poland

*Corresponding author: Jagiellonian University, Institute of Psychology, Pain Research Group, ul. Ingardena 6, 30-060, Kraków, Poland. E-mail address: karolina.wiercioch@uj.edu.pl


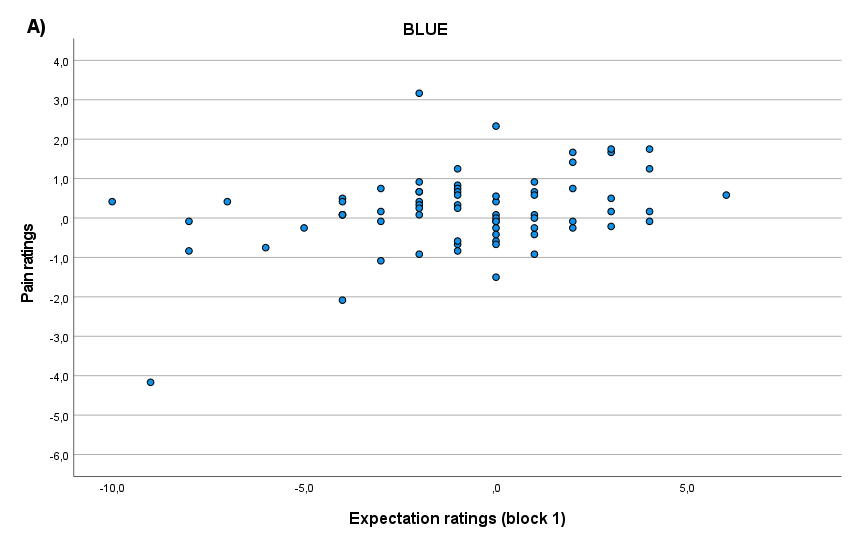


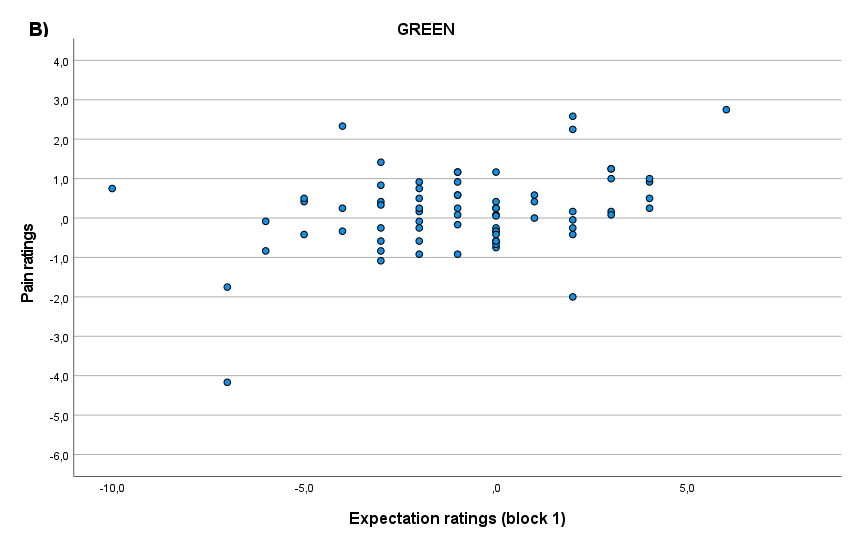


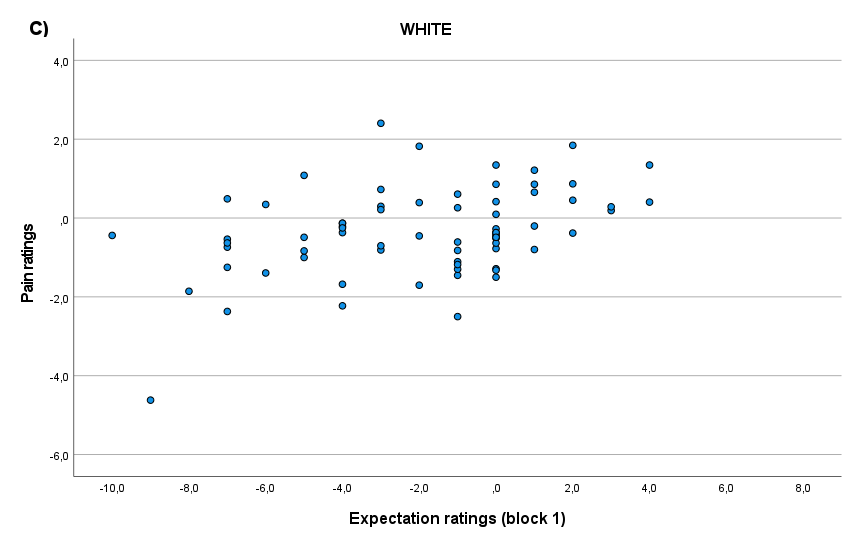


Figure S1. Relationship between expectation ratings from the first expectation block and pain intensity ratings, for blue (A), green (B) and white (C).
